# Automatic Detection and Characterization of Autonomic Dysreflexia Using Multi-Modal Non-Invasive Sensing and Neural Networks

**DOI:** 10.1089/neur.2022.0041

**Published:** 2022-11-10

**Authors:** Shruthi Suresh, Thomas H. Everett, Riyi Shi, Bradley S. Duerstock

**Affiliations:** ^1^ Weldon School of Biomedical Engineering, Purdue University, West Lafayette, Indiana, USA.; ^2^ Krannert Cardiovascular Research Center, Indiana University School of Medicine, Indianapolis, Indiana, USA.; ^3^ Center for Paralysis Research, West Lafayette, Indiana, USA.; ^4^ Department of Basic Medical Sciences, West Lafayette, Indiana, USA.; ^5^ School of Industrial Engineering, West Lafayette, Indiana, USA.

**Keywords:** autonomic dysreflexia, electrophysiology, rat, routine physiological monitoring, spinal cord injury

## Abstract

Autonomic dysreflexia (AD) frequently occurs in persons with spinal cord injuries (SCIs) above the T6 level triggered by different stimuli below the level of injury. If improperly managed, AD can have severe clinical consequences, even possibly leading to death. Existing techniques for AD detection are time-consuming, obtrusive, lack automated detection capabilities, and have low temporal resolution. Therefore, a non-invasive, multi-modal wearable diagnostic tool was developed to quantitatively characterize and distinguish unique signatures of AD. Electrocardiography and novel skin nerve activity (skNA) sensors with neural networks were used to detect temporal changes in the sympathetic and vagal systems in rats with SCI. Clinically established metrics of AD were used to verify the onset of AD. Five physiological features reflecting different metrics of sympathetic and vagal activity were used to characterize signatures of AD. An increase in sympathetic activity, followed by a lagged increase in vagal activity during the onset of AD, was observed after inducing AD. This unique signature response was used to train a neural network to detect the onset of AD with an accuracy of 93.4%. The model also had a 79% accuracy in distinguishing between sympathetic hyperactivity reactions attributable to different sympathetic stressors above and below the level of injury. These neural networks have not been used in previous work to detect the onset of AD. The system could serve as a complementary non-invasive tool to the clinically accepted gold standard, allowing an improved management of AD in persons with SCI.

## Introduction

Autonomic dysreflexia (AD) is a unique manifestation in persons with spinal cord injury (SCI) above the T6 (thoracic) level.^1,2^ AD is characterized clinically by an acute increase in systolic blood pressure (SBP) of at least 20 mm Hg and sudden, uninhibited sympathetic discharges and may be accompanied by bradycardia. It is a potentially life-threatening condition commonly initiated by irritation or noxious stimuli below the level of injury or triggers, with 85% of cases of AD being triggered by urinary tract infections or impacted bowels.^3^ AD causes debilitating symptoms above the level of injury^4^ and can cause a dangerous increase in blood pressure (BP) if left untreated.^5,6^ In a study of life-threatening instances of AD, 22% of cases resulted in death.^7^ However, being familiar with the symptoms and triggers of AD remains the standard approach for managing AD for newly injured tetraplegics.^8,9^

Only 41% of persons with SCI or their family had heard of AD, even though 22% of persons with SCI reported symptoms consistent with unrecognized AD.^10^ Meticulous monitoring of telltale symptoms of AD can prevent the rapid escalation of AD-induced hypertension and reduce risks to personal health if managed quickly. However, learning to recognize AD symptoms can take time and identifying the source of noxious stimuli occurring in paralyzed parts of the body may be difficult^11^ to the detriment of the person, especially for those who are newly adapting to living with paralysis. A non-invasive, continuous monitoring device is needed to readily detect instances of AD, particularly for asymptomatic or “silent” AD episodes, which can be potentially harmful to cardiovascular end-organs because of recurrent episodes of paroxysmal rises in BP without concomitant symptoms.^12^

Clinically, medical professionals use BP monitoring to diagnose AD.^13^ However, this method is impractical for continuous monitoring for the presence of AD for long-term use given that it restricts persons' activities and can be affected by movements such as wheeling or transferring. The tactile and sonorous stimuli to measure BP can be distracting, interrupting activities of daily living (ADLs) or sleep.^14^ Moreover, the sampling rate of measuring BP is quite low, at most every 5 min, when using ambulatory blood pressure monitoring systems (ABPMs). Interpretation of the ABPM data requires a trained clinician and typically a computer and data processing software as well as the required training on ABPM usage, data analysis, and interpretation. This hinders widespread adoption of AD monitoring in the SCI community.^15^ There is a need for a sensitive, yet non-invasive, method of detecting the onset of AD that can be adopted easily into clinical practice and for at-home use.

The development of AD in response to SCI has been investigated in human^5,16^ and animal models.^17,18^ Rat models in particular have been used by other researchers to study the onset of AD. These studies often focus on comparing pharmacological interventions when severe AD occurs as well as the mechanism of the onset of AD.^19–21^ However, few researchers have explored the unique physiological signatures of AD using a multi-modal array of sensors that is not dependent upon a significant increase in BP. These studies rely entirely on the pre-determined patterns in BP measured by a telemetry system to identify the onset of an AD event induced by a trigger.^22,23^ However, there are no studies known to us that have trained machine learning algorithms to automate the process of detecting AD during onset using non-symptom-based approaches. In this article, we explore the use of multi-modal, wearable sensor technologies to characterize the unique signatures of AD from different sympathetic stressors and the use of machine learning models to automate the process using a rodent model of SCI.

## Methods

### Animals

Nineteen male Sprague-Dawley rats were used for this study. All animals were between 3 and 5 months of age and weighed 450–600 g post-injury. Animals were provided food and water *ad libitum* post-injury and were presented with treats to enrich their diets.^24^ Experiments were performed in accordance with the international directions for the protection of animals used for scientific purposes, and the protocol was approved by the Purdue University Institutional Animal Care and Use Committee.

### Rat spinal cord injury and post-operative care

Animals were given compression SCIs, as previously described, using forceps with a détente at the T2/T3 level.^25,26^ Injury was verified through visual observation of damage to the spinal cord determined by the ischemia and clotting patterns observed in the spinal cord. To validate functional loss after SCI, motor and sensory responses in hindlimbs were evaluated through toe-pinch. Animals were also observed after surgical recovery on an open tabletop to gauge their ability to support body weight and use the hindlimbs for movement. Rats received multi-modal analgesia—buprenorphine (0.1 mg/kg, subcutaneous [s.c.]) two times a day and meloxicam (1–2 mg/kg s.c.) one time a day for 3 days post-surgery with regular bladder expression. The health of rats was monitored using established veterinary medicine protocols.^24^

### Data acquisition

**FIG. 1.**
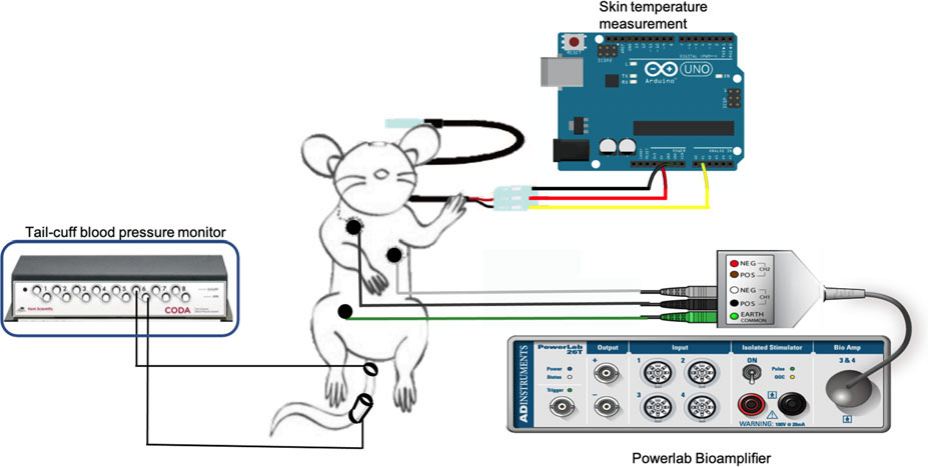
Schematic of the sensors. Non-invasive electrodes placed on the ventral skin surface of a rat in Lead I configuration, the Coda^®^ Blood Pressure system with occlusion and VPR cuff, and a temperature probe connected to an Arduino^®^. VPR, volume pressure recording.

Data were collected from an array of cutaneously attached sensors developed in past work in animals acclimated to the sensor array.^27^ Non-invasive sensors measured electrocardiography (ECG), BP, skin temperature, and skin nerve activity (skNA), which measures nerve activity of the stellate ganglion,^28^ from awake, restrained animals (Fig. 1). ECG and skNA were collected at a sampling rate of 10 kHz. BP was collected every 30 sec using a tail-cuff monitoring system. Skin temperature was recorded at a rate of 2 samples/sec.

Rodents were restrained using plastic holders with air holes and a darkened nose cone, as used for tail-cuff measurements (HLD-RL model; Kent Scientific Corporation, Torrington, CT). In order for the rats to not be stressed during physiological recording, we developed a 4-week protocol to acclimate the rats to being restrained with the non-invasive sensor array attached cutaneously. Before colorectal distension (CRD) experimentation, rats had acclimated to the sensor restraint inasmuch as the average SBP and heart rate (HR) did not increase >3% before being introduced to the restraint system to when experiments began (unpublished data).

AD was induced in rats using CRD produced by inflating a balloon inserted in the rectum, with 2 mL of air for a period of 1 min.^17,29^ Three distensions were produced on each day of testing with a 10-min recovery period. CRD was produced on days 7, 9, 11, and 14 post-injury. A CRD event was considered as successfully inducing AD if it led to a rapid increase of SBP by at least 15 mm Hg. All increases in SBP were preceded by spikes in sympathetic activity and followed by bradycardia. There also was an increase in mean arterial pressure (MAP) of at least 10 mm Hg.^21,23,30^ Positive AD events were used for training, testing, and validating the NeuralNet described in the following section.

Additional sympathetic stress responses were induced above the level of injury through an acoustic startle, with 1 min of intermittent startle at 108–112 dB.^27^ The nociceptive stimulus below the level of injury was induced through a persistent tail pinch. The tail pinch was stimulated by a weight of 700 g applied to the tail of the rat for a duration of 1 min. Order of the stimuli was randomized using a randomizer algorithm to reduce potential order effects.

### Neural network development

Time-series signals were filtered to remove motion artifacts and other high-frequency noise.^35^ Thirty-six relevant physiological features were extracted using 15-sec windows of the different sensor data.^31^ These comprise well-established cardiovascular and neural features in the ECG and microneurography literature,^32,33^ which reflect sympathovagal discharge. Extracted features were normalized using the min-max scaling method using data from the same trial day.

Using feature selection techniques, we determined five features that were highly significant and relevant in the characterization of AD.^31^ These five features represented changes in both the sympathetic and vagal branches. They included number of bursts of sympathetic nerve activity, average value of the integrated skNA (iskNA), median value of the normal to normal (NN) intervals—medianNN, the root mean square of successive differences (RMSSDs), and the percentage of number of NN intervals >5 ms (pnn5). Number of bursts and iskNA are representative of sympathetic activity whereas medianNN, RMSSD, and pnn5 are reflective of vagal activity.

In the article,^35^ we also explored various statistical and machine learning techniques and identified the neural network (NeuralNet) to be the best performing. In this article, we further refine upon that model and trained a five-layer NeuralNet with a rectified linear unit activation function using the data (five neurons in the input layer, one neuron in the output layer; hidden layers neurons were calculated by the sklearn algorithm). A total of 2200 data points were collected from animals. We split the data into three stratified sets—the training set (70%), the test set (15%), and the validation set (15%), and 10-fold cross-validation was used to gauge the performance of the NeuralNet.

### Statistical analysis

A *t*-test (*p* < 0.01) was used to determine significant differences in the features during AD and non-AD events. An analysis of variance (ANOVA; *p* < 0.01) was used to determine significant differences between responses attributable to the other sympathetic stimuli. A chi-squared test was used to determine which features most closely resulted in changes attributable to the onset of AD and other sympathetic reactions attributable to the stressors. Outliers were determined as those that had values >3 standard deviations away from the mean and were removed.

We measured the accuracy, sensitivity (true positive rate), specificity (true negative rate), F-1 score, and area under the curve (AUC)/receiver operating characteristic (ROC) of the developed NeuralNet.

## Results

### Characteristics of autonomic dysreflexia through signature changes

**FIG. 2.**
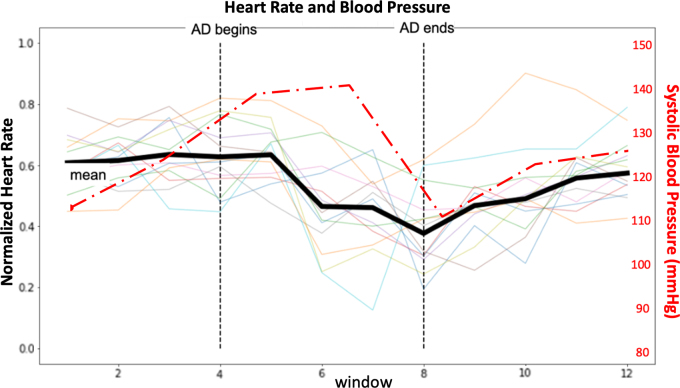
Significant decrease in mean normalized heart rate (black solid trace) and increase in representative systolic blood pressure (red dot-dash trace) over the course of the AD event (indicated by the vertical dotted lines) as a result of the sympathovagal response. After the stimulus was removed, heart rate and blood pressure returned to pre-AD levels. *N* = 13. AD, autonomic dysreflexia.

As anticipated, a statistically significant decrease in heart rate over the course of the AD event was observed (Fig. 2). There was also an average increase in SBP by 19.8 mm Hg, an increase in diastolic BP by 13.9 mm Hg, and an increase in MAP by 15.2 mm Hg during the AD event (Table 1). Overall, 91 of the 130 (70.6%) instances of CRD recorded from rats were considered AD; these translated to 1540 data points, which were used for the training of the NeuralNet.

**Table 1.** Changes in Blood Pressure Attributable to the Various Stimuli

Stimulus	Δ Systolic Blood Pressure (mm Hg)	Δ Diastolic Blood Pressure (mm Hg)	Δ Mean Arterial Pressure (mm Hg)
AD	+19.8^*^	+13.9^*^	+15.2^*^
Tail-pinch	+5.5	+0.4	+0.9
Startle	+7.1^*^	+1.0	+2.1

Largest changes were observed as attributable to AD.

^*^ Indicates significant difference (*p* < 0.01) compared to baseline values.

AD, autonomic dysreflexia.

**FIG. 3.**
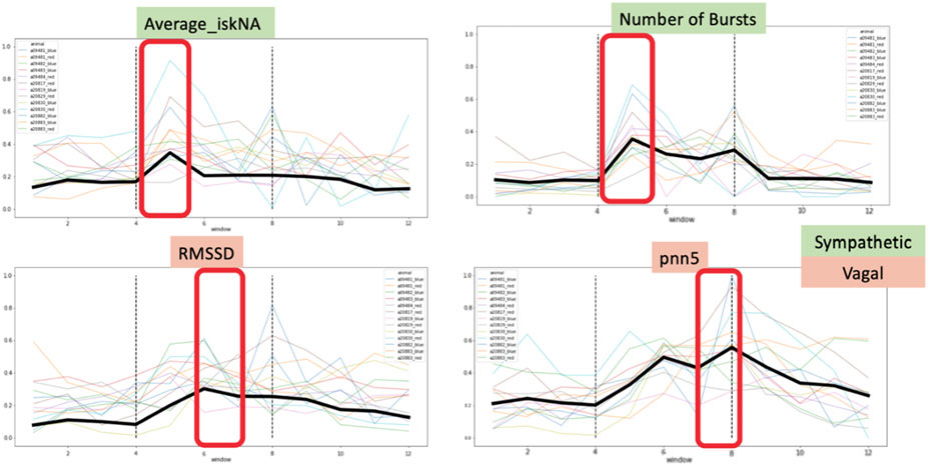
Increase in sympathetic activity (average iskNA and number of bursts) precedes the increase in vagal activity (RMSSD and pnn5) after CRD. The y-axis is the normalized units of each feature whereas the x-axis represents windows of 15 sec each. Color legends indicate signatures of individual rats. Bold black line in each chart represents mean values of *N* = 13 rats. CRD, colorectal distension; iskNA, integrated skin nerve activity; pnn5, percentage of number of NN intervals >5 ms; RMSSD, root mean square of successive differences.

In addition to these well-established metrics of AD detection, we observed a statistically significant increase from baseline during AD in all five aforementioned features representing sympathetic and vagal activity. We were able to quantify the cascade of sympathetic and vagal activity on a time-scale resolution of 15 sec (Fig. 3) through our sensors. We observed an initial increase in sympathetic activity elicited by the onset of AD (dashed lines in Fig. 3), characterized by the increase in values of the average iskNA and the number of bursts detected through the skNA sensors. This was followed by a delayed increase in vagal activity characterized through the increase in the RMSSD and pnn5 features toward the end of the AD episode.

### Varying signatures attributable to different stimuli

When compared to baseline (non-stimulus) values of SBP, both AD and acoustic startle responses led to significant (*p* < 0.05) increases, but tail-pinch did not lead to a statistically significant increase (Table 1).

**FIG. 4.**
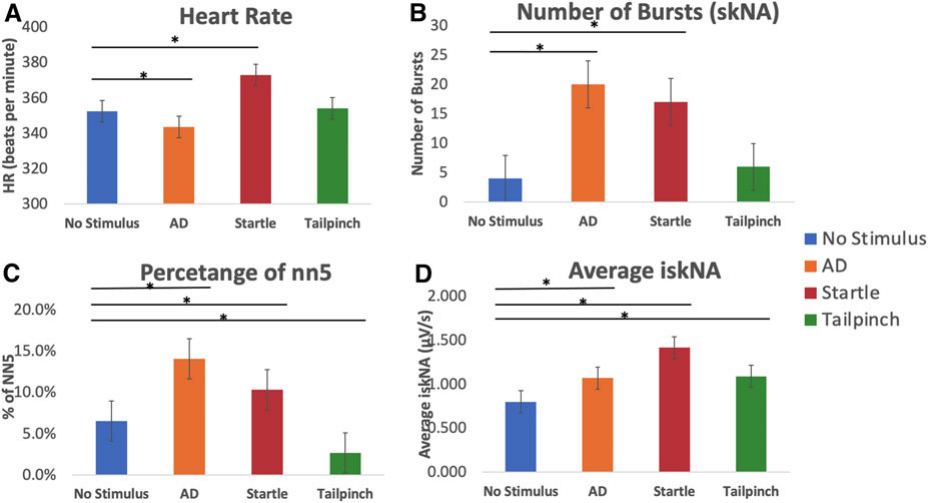
Changes in median values from non-stimulus of (**A**) heart rate, (**B**) number of bursts per minute, (**C**) pnn5, and (**D**) average iskNA attributable to the different stimuli. A one-way ANOVA was performed for the ANS activity features (pnn5, average iskNA, and number of bursts) across the three stimuli. *Indicates significant difference (*p* < 0.01) from baseline values. AD, autonomic dysreflexia; ANOVA, analysis of variance; ANS, autonomic nervous system; iskNA, integrated skin nerve activity; pnn5, percentage of number of NN intervals >5 ms.

In Figure 4A, we observed a statistically significant decrease in HR compared to baseline activity (*p* < 0.01). However, HR was significantly increased during the startle response (*p* < 0.01). There was no significant difference in changes in HR during tail-pinch compared to no stimulus.

When compared to baseline values, all three stimuli led to an increase in sympathetic activity characterized by an increase in average iskNA (Fig. 4D) and number of bursts with AD and startle compared to no stimulus and tail-pinch (*p* < 0.01; Fig. 4B).

There was a statistically significant increase from baseline values in vagal activity attributable to AD and startle (*p* < 0.01), except for tail-pinch, which exhibited a statistically significant decrease in pnn5 (*p* < 0.01; Fig. 4C).

### Discriminating the different stimuli using machine learning

The five-layer NeuralNet developed using the data was able to distinguish AD from non-AD events with an accuracy of 93.4%, a sensitivity of 93.5%, and a specificity of 93.3%. Additionally, it also had an AUC-ROC of 0.93, suggesting that the NeuralNet was able to distinguish between the two classes with a high level of confidence.

**FIG. 5.**
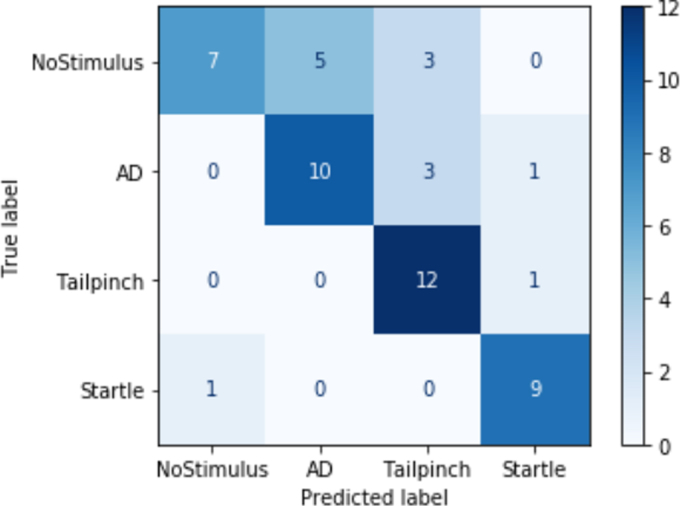
Confusion matrix of the multi-class neural network classifier trained on the data from the different stimuli. Tail-pinch and startle data were least likely to be misclassified as another class, whereas non-stimulus data were most likely to be misclassified. AD, autonomic dysreflexia.

When trained on the multi-stimulus data set with the three selected stimuli (i.e., CRD, acoustic startle, and tail-pinch), the NeuralNet had a weighted average performance of 79% accuracy. However, it performed the best on the detection of tail-pinch data with a precision of 0.91 whereas non-stimulus data were more likely falsely classified leading to more false positives (Fig. 5).

## Discussion

Recognition and prevention of AD-related signs and symptoms play a critical role in preventing their escalation to more dire medical conditions. Currently, the standard approach for managing AD is to train persons with SCI to recognize their symptoms and promptly alleviate the AD trigger, which can be difficult to identify and frequently requires the assistance of a caregiver. There is a need for a sensitive, yet non-invasive, method of detecting the onset of AD, which can be adopted easily into clinical practice and for at-home use.^15^ We propose a method that permits continuous monitoring that could inform persons with SCI of the onset of AD before extreme hypertension occurs.

We also demonstrate a method of distinguishing different types of sympathetic stressors, including nociceptive pain and startle, from AD that could play an important role in reducing the onset of false positives as a result of sympathetic activation. This could help improve the performance of the machine learning model by reducing the incidence of false positives attributable to sympathetic hyperactivation. In addition, the ability to discriminate between different sympathetic stressors would also prove to be valuable in assisting the management of AD by identifying possible triggers.

In this article, we demonstrated the following: 1) The onset of AD can be characterized through the concomitant cascade of sympathovagal discharge using an array of non-invasive sensors; 2) a neural network can be trained to distinguish between AD and non-AD events with a 93.4% accuracy; and 3) differentiate CRD from other sympathetic stressors with a 79% accuracy.

### Characterizing sympathovagal signatures of autonomic dysreflexia through non-invasive sensing

The sympathovagal cascade attributable to the onset of AD is well understood in clinical literature.^37,38^ All signs and symptoms of AD are attributed to the hyperactivity of the sympathetic impulses below the level of injury and the compensatory vagal activation above the level of injury.^2^ However, there is no tool that characterizes the resultant physiological cascade from the onset of AD through multiple sensing modalities.

The combination of non-invasive, wearable ECG and skNA sensors allowed us to measure sympathetic and vagal coactivation during the onset of AD. The initial increase in sympathetic activity within seconds of introduction of the trigger is followed by parasympathetic activity toward the end of the AD episode. This signature, coupled with the anticipated bradycardia and increase in BP, provides a quantitative understanding of the sympathovagal cascade, which occurs during the onset of an AD episode. However, our BP measurement method did not monitor beat-by-beat changes. Because of our non-continuous method of BP measurement using tail-cuff monitoring, we likely missed capturing many SBP spikes during CRD. However, we were able to verify AD episodes in ∼71% of CRD events. Through features extracted from the ECG and novel skNA sensors, we characterized AD on a greater temporal resolution than currently available clinically through stand-alone measurements of BP through ambulatory BP systems. Arterial BP telemetry devices may be used in the future to track hemodynamic changes in real time.^17^

Sympathetic activity was characterized principally by changes detected through the skNA sensors, which has been extensively validated as a non-invasive surrogate for stellate ganglion nerve activity.^28,30^ The stellate ganglion is known to be an important source of cardiac sympathetic innervation and also gives rise to sympathetic nerves, which innervate blood vessels and sweat glands in the skin.^35,36^ Sympathetic pre-ganglionic fibers originating from T5-T9 spinal cord levels bypass the paravertebral ganglia and synapse onto post-ganglionic neurons in the stellate ganglion.^37^ Increases in average value of iskNA suggest an increase in sympathetic tone during the onset of AD. The increase in the number of spikes/bursts over the duration of AD assesses the transient increase in synchronous firing of the stellate ganglion.^38^ The sympathetic discharge occurs within 15 sec of the introduction of the noxious trigger. However, one of the limitations of the skNA sensor is the inability to gauge parasympathetic activity.^39^

ECG data have been used extensively to determine vagal tone.^40^ Toward the end of the AD episodes, a sharp increase is noted in the RMSSD and pnn5. Both these metrics are reliable indices for parasympathetic activity for short-term measurements and are strongly correlated with spectral components of the ECG. RMSSD is a reflection of beat-to-beat variance in HR and often estimates the vagally mediated changes reflected in HR variability.^41^ Similarly, pnn5 is closely related to vagal activity.^42^

### Distinguishing multi-stimulus data

All stimuli led to an increase in sympathetic activity detected by the sensors. CRD and the startle response resulted in concomitant activation of the sympathetic and vagal systems. CRD resulting in AD causing a major jump in BP, whereas HR decreased overall. In comparison, the acoustic startle response, a sympathetic trigger above the level of injury, led to a higher sympathetic activation resulting in increased HR and average iskNA. The observed startle response is in agreement with past research that shows hypersensitivity to the acoustic startle reflex in persons with SCIs.^43–45^ This is likely the result of the neuroplasticity observed post-injury wherein there is reorganization after SCI at the cortical as well as brainstem levels.^46^

After tail-pinch, we observed a significant increase in average iskNA, but not in the number of bursts; nor did we observe significant changes in BP and HR after the nociceptive stimuli attributable to the interruption of supraspinal innervation post-SCI. Though tail-pinch has been used in past research in mice as a tool to induce AD,^47^ in our experiments, we did not observe a resultant increase in BP to characterize the response as AD according to our criteria. However, the anticipated sympathetic surge in the tail-pinch response was accompanied by a concomitant vagal inhibition response.^48^ It may be possible that the crush injury that induced the SCI in our rats may have led to incomplete injuries.

### Machine learning as a tool to characterize autonomic dysreflexia

The NeuralNet created using the data set was able to discern the differences between AD and non-AD data with an overall high performance across all the relevant metrics. The use of BP changes as the ground truth led to validation of this model. We prioritized the development of a model that was accurate and has a high sensitivity and specificity. A balance between the sensitivity and specificity in the detection of AD ensures low false-negative rates. A high false-negative rate wherein the model did not detect AD when it was occurring could lead to serious medical consequences. In contrast, the incidence of false-positive errors would be more of an inconvenience to users rather than not detecting episodes of AD at all. The model's high AUC-ROC (0.93) demonstrated its ability to detect the onset of AD symptoms with high accuracy with an inclination toward low false-negative rates. The strong performance of the neural network as a tool to characterize AD also validates the trends observed in the temporal data collected from the sensor data.

## Conclusion

Current methods to detect AD are not feasible for long-term monitoring. Through this article, we have presented a tool that enables rapid detection of AD. Moreover, we were able to study the onset of AD on a higher temporal resolution, allowing the determination of a unique signature through changes in the sympathovagal branches of the autonomic nervous system (ANS) during AD. Last, the use of machine learning enables an automated detection of the onset of AD distinguished from other sympathetic triggers.

A non-invasive sensor system capable of detecting the onset of AD can improve the independence and quality of life of persons with an SCI. Additionally, the implementation of the early detection system could allow persons with more time to identify and eliminate the trigger before escalation to dangerous hypertensive levels. The innocuous nature of many precipitants of AD often makes it difficult to avoid the occurrence of AD. Tetraplegics experienced AD ranging from several times a day^49^ to a few times in a month. By combining the AD detection system with wireless mobile connectivity, caregivers can be automatically alerted when AD is occurring. This would enable tetraplegics greater autonomy to work, go to school, and participate in their communities, knowing that if an AD episode occurs, there will be emergency oversight.

## Abbreviations Used

ABPMs: ambulatory blood pressure monitoring systems
AD: autonomic dysreflexia
ADLs: activities of daily living
ANOVA: analysis of variance
ANS: autonomic nervous system
AUC: area under the curve
BP: blood pressure
CRD: colorectal distension
ECG: electrocardiography
HR: heart rate
iskNA: integrated skNA
MAP: mean arterial pressure
NeuralNet: neural network
NN: normal to normal
pnn5: percentage of number of NN intervals >5 ms
RMSSDs: root mean square of successive differences
ROC: receiver operating characteristic
SBP: systolic blood pressure
s.c.: subcutaneous
SCI: spinal cord injury
skNA: skin nerve activity
